# A Framework for Comprehensive Dairy Calf Health Investigations

**DOI:** 10.3390/ani15020181

**Published:** 2025-01-11

**Authors:** Kristen Y. Edwards, David L. Renaud

**Affiliations:** Department of Population Medicine, University of Guelph, Guelph, ON N1G 2W1, Canada; kedwar01@uoguelph.ca

**Keywords:** colostrum, diarrhea, heifer, preweaned, respiratory disease, veterinary

## Abstract

The objective of this narrative review is to provide a systematic framework for veterinarians to investigate dairy calf health, focusing on critical control points and key performance indicators (KPIs) to address morbidity and mortality challenges in preweaned calves. Recommendations target prenatal maternal nutrition, heat stress abatement, and optimal calving management to minimize risks associated with perinatal mortality and preweaning morbidity. Further, comprehensive colostrum management is discussed to ensure excellent transfer of passive immunity, which includes prompt collection and feeding within two hours of birth at a volume of 8.5–10% of calf body weight. Nutritional guidance emphasizes the importance of transition milk and feeding higher planes of nutrition to support immunity, with recommendations that milk total solids exceed 10% to meet energy needs. Environmental management recommendations include a minimum of 3.3 m^2^ of space per calf, the use of low-dust bedding, and air quality controls to reduce respiratory disease. Lastly, regular health data collection and KPI monitoring, such as average daily gain and morbidity rates, are essential for data-driven improvements. By implementing these evidence-based recommendations, veterinarians can support dairy farmers in reducing calf morbidity and mortality, ultimately enhancing calf welfare and lifetime productivity.

## Simple Summary

Over one-third of calves will experience a preweaning illness, with neonatal calf diarrhea and respiratory disease accounting for the majority of preweaning diseases and deaths. This narrative review offers a structured framework for veterinarians to investigate dairy calf health management challenges by addressing critical control points, monitoring key performance indicators, and assisting thorough calf health record-keeping by producers. Key areas in calf health assessments include prenatal and perinatal factors such as maternal nutrition, heat stress, and calving management practices. Additionally, essential topics such as colostrum management, preweaning nutrition, housing, environmental conditions, and hygiene practices are discussed. By adopting a comprehensive approach, veterinarians can provide tailored recommendations to dairy producers to reduce disease incidence and improve calf growth and future productivity. This review highlights the value of data-driven insights and routine evaluations to enhance calf health outcomes on dairy farms.

## 1. Introduction

Raising heifers represents one of the largest expenses on dairy farms [1] and can account for up to 20% of total annual milk production costs [2]. However, a quarter of female calves born on the dairy will either never make it to the lactating herd [3] or they will experience illnesses that reduce their genetic potential for milk production [4,5]. Further, over one-third of dairy calves will experience a preweaning illness, with neonatal calf diarrhea (NCD) and bovine respiratory disease (BRD) accounting for the majority of recorded morbidities and mortalities [6]. Specifically, digestive disorders account for 56% of preweaning morbidities and 32% of recorded mortalities, and BRD is responsible for 33% of morbidities and 14% of mortalities [6]. One of the primary reasons for high morbidity and mortality rates in preweaned calves is that they are unable to mount effective immune responses early in life [7] and rely on the transfer of passive immunity (TPI) for their immune defenses [8]. With the detrimental effects of morbidity and mortality, it is critical to reduce preweaning morbidity and mortality to improve both welfare and profitability.

Veterinarians serve as key advisors on dairy farms; thus, the objective of this narrative review is to provide veterinarians with a systematic approach for investigating dairy calf health challenges by identifying critical control points (CCPs), monitoring key performance indicators (KPIs), and assisting with calf health record completeness.

## 2. Materials and Methods

Google Scholar and Web of Science databases were searched in October and November 2024 for scientific articles. The following search terms were used alone or in combination: “calves” OR “calf”, “preweaned” OR “preweaning”, “dairy”, “bovine respiratory disease” OR “BRD”, “diarrhea” OR “scours”, “morbidity”, “mortality”, (“prepartum” OR “late gestation” OR “dry cow*”), (“immune” OR “immunity”), (“heat stress” OR “heat abatement“), “colostrum”, (“milk replacer” OR “milk”), “transition milk”, (“starter OR grain”), “water”, (“air quality” OR “ventilation”), “health record*”, “automated milk feeder*”, (“thoracic ultrasound” OR “lung consolidation”), and (“vaccination*” OR “vaccine*”). Articles were manually screened by title and abstract to exclude manuscripts that did not describe investigations into calf health management or the impacts of late gestation on calf health and survivability. The full articles were then assessed for their inclusion eligibility, with case reports and case series studies being excluded. To identify additional relevant literature, the references cited within key manuscripts were also reviewed. This approach allowed for the discovery of additional sources that contributed to the development of a comprehensive understanding of the topic. In total, 171 references were included in the final narrative review.

## 3. Prenatal Factors

During late gestation, the bovine fetus experiences significant growth and proliferation of immune cells [9], and exposure to stressors can adversely affect the developing immune system. Important areas to consider when conducting calf disease investigations include late gestation nutrition, heat stress, and the calving pen environment.

### 3.1. Maternal Nutrition

Restricted energy supply of the late gestation dam can result in lowered humoral responses to vaccination in calves [10], as well as a 10-percentage-point increase in calf morbidity [11] and a 5-percentage-point increase in calf mortality [12]. While the association between maternal overnutrition and calf morbidity and mortality has not been as thoroughly studied, excessive prepartum energy density increases fetal birthweight and risk for perinatal mortality [13,14].

Assessing formulated rations for metabolizable energy (ME), metabolizable protein (MP), and predicted vs. actual dry matter intakes (DMIs) can ensure that rations meet late gestation requirements (Table 1). It is also important that the mature cow bodyweight (MBW) used in ration formulation is based on each individual herd’s MBW, as it can vary by 170 kg between herds [15]. The MBW is defined as the bodyweight of cows in their third lactation or greater, excluding cull cows, and is measured at consistent days in milk (DIM), ideally between 80 and 200 DIM [16]. Weigh tapes can be used to determine MBW, as they are reasonably accurate in cattle over 150 kg with <5% difference between the true bodyweights and SD-adjusted bodyweights [17].

Beyond evaluating the formulated ration, assessing feed availability (e.g., number of feed push-ups per day, amount of time the bunk is empty, frequency of feed delivery, and bunk space per cow) and calculating dry matter intakes to determine if they match the formulated intakes will help to ensure that cows are consuming the ration as intended. Sorting should also be assessed by using a four-screen Penn State Particle Separator (PSPS) [18,19] on freshly delivered feed and refusals. Sorting of each PSPS fraction can be calculated by dividing the actual amount of feed consumed by the predicted amount and expressing it as a percentage for each PSPS fraction, where >100% suggests preferential consumption and <100% suggests selective refusals [20]. While there is no scientific consensus regarding sorting cut-points, <10% of long particles being refused has been considered to be indicative of minimal sorting based on changes to rumen pH [21]. If sorting is identified, decreasing the dry matter of the ration to 45% can minimize sorting against long particles and improve intake [22]. Additionally, ensuring that particle length does not exceed 2.54 cm can further reduce sorting [23].

**Table 1:** Nutrient recommendations ^a^ for far-off and close-up dry Holstein cows.

Variable	Far-Off Dry Cows	Close-Up Dry Cows
Dry matter intake (kg/d)	13–14	~13
Metabolizable energy (Mcal/kg)	1.8–2.0	2.1–2.2
Crude protein (% DM)	<16	<15
Metabolizable protein (MP; g/d)	1000–1100	1200–1400
Methionine (% of MP)	No guideline	2.6–2.8
Lysine (% of MP)	No guideline	6.8–7.0
Starch (% DM)	<13	16–18
Non-fiber carbohydrate (NFC; % DM)	20–30	30–34
Total fat, optimum (% DM)	3.5	3.5
Calcium (% DM)	0.5–0.7	1.5
Phosphorus (% DM)	0.3–0.35	0.3–0.35
Magnesium (% DM)	0.2–0.25	0.45–0.50
Potassium (% DM)	<2.0	<1.3
Sodium (% DM)	0.1–0.2	0.1–0.2
Chloride (% DM)	0.4–0.8	0.4–0.8
Sulfur (% DM)	0.3	0.3
Selenium ^b^ (mg/kg)	0.3	0.3
Zinc ^c^ (mg/kg)	60–80	60–80
Vitamin A (IU)	75,000	75,000
Vitamin D (IU)	25,000	30,000
Vitamin E (IU)	500	1800

^a^ Adapted from Overton et al. [24]. ^b^ FDA limit for added selenium. ^c^ Total diet concentration.

### 3.2. Heat Stress

Late-gestation heat stress has a profound impact on the developing bovine fetus. Heat-stressed cows give birth to lighter calves, in part due to decreased DMI during heat stress [25]. Further, calves born to heat-stressed dams have altered immune competencies and reduced serum IgG concentrations [26], and increased removal from the herd prior to puberty due to illness or poor growth [27].

Late-gestation heat stress can be assessed by evaluating the respiration rates of dry cows. Respiration rates of >61 breaths per minute are an indication of heat stress in dry cows [28]. Additionally, respiration may be monitored using accelerometry tags to detect heavy breathing as an indication of heat stress in scenarios where manually assessing respiration rates is impractical [29]. When heat stress is detected, heat abatement techniques, including providing shade, fans, and sprinklers, have been shown to effectively reduce heat stress in dry cows [30]. Using a combination of fans and sprinklers may be the most effective approach to lower body temperature and reduce respiration rates in cows [31]. Research suggests that sprinkler flow rates of 1.3 L/min are sufficient to provide cooling [32], with longer soak times (20 to 30 s) proving more effective than shorter durations (10 s) [33]. Additionally, fans should be directed toward the resting area, delivering air speeds of at least 1 m/s at the cows’ resting height for optimal cooling [34]. Fan spacing will depend on type and size, and using an anemometer can assist in making fan spacing recommendations [35]. Lastly, water accessibility is crucial for heat-stressed cows. As heat stress increases, cows spend more time at waterers, drink more frequently, and consume greater quantities of water per day [36]. Conversely, water deprivation can enhance the effects of heat stress [37].

## 4. Calving Management

Perinatal mortality refers to the death of a calf before, during, or within the first 48 h after calving [38], and is estimated to have a rate of 6.2% worldwide [39]. Reviewing calving records can help identify whether perinatal mortality is a concern, highlight which parities are most affected, and uncover potential causes.

Dystocia is the primary cause of perinatal mortality [39], and calves that survive dystocia have increased odds for BRD and NCD [40]. Primiparous animals are at the highest risk of dystocia [41]. Ensuring that heifers reach an appropriate body weight at breeding (55% of MBW) and targeting a body condition score (BCS) of 3.0–3.5 [42] to avoid over-conditioning (BCS ≥ 4.0) [43] can help reduce the risk of dystocia in this group. Additionally, dystocia is largely driven by heavier calf birthweights [44], thus assessing the calving ease of selected sires, as well as incorporating beef [45] and sexed semen [40], can help minimize issues with feto-maternal disproportion by reducing calf birthweights. Additional management factors associated with dystocia include moving cattle during stage I of parturition (beginning of cervical dilation and increased restlessness), as it results in prolonged stage II labor [46] and increased risk of stillbirths [47]. Lastly, calving supervision and timely intervention are critical for reducing perinatal mortality. Evidence shows that, for each additional hour beyond 2 h in stage II of parturition, the odds of stillbirth increase by 30% [47]. Furthermore, assisting cows showing no signs of progress ~70 min after the onset of stage II has been shown to significantly reduce stillbirths [48]. Frequent analysis of records and provision of calving management standard operating procedures (SOPs) can aid in the reduction of perinatal mortality and should be provided for dairy producers. Only 60% of operations in the United States have guidelines on when to intervene during calving [49]. Further, while little is known about how commonly calving management SOPs are provided by veterinarians, one Canadian study reported that 55% of farms requested that veterinarians be part of general SOP development [50]. This highlights an opportunity for veterinarians to take a more active role in assisting farms with the development and implementation of calving management SOPs to reduce the risk of perinatal mortality.

## 5. Perinatal Care

The risk of perinatal mortality extends beyond dystocia and is influenced by the quality of perinatal care. Preventing neonatal hypothermia is essential, as it plays a role in reducing the likelihood of failed transfer of passive immunity (FTPI) and perinatal mortality [51] Hypothermia is a risk to calves whose rectal temperatures are below 37 °C immediately after birth [51] and, while calves exposed to colder ambient temperatures are at higher risk for hypothermia, it is also influenced by other factors, including dystocia [52]. Prevention strategies include rapid administration of colostrum for caloric support to maintain a thermoneutral state [53], drying of the newborn to support thermostability [54], and the use of forced air warming boxes [55].

Additional perinatal care also includes assessing the vitality of the newborn calf to identify high-risk calves and implement interventions. Vitality can be assessed using the VIGOR scoring system for dairy calves [56]. Calves identified as high risk using the VIGOR scoring system may benefit from oxygen therapy, artificial respiration, buffer therapy for acidosis [51], or meloxicam administration [56].

The prevention of umbilical infections in newborn calves is also important, as they are associated with an increased risk of mortality [57]. Despite the common practice of umbilical disinfection, little research exists to support its practice. For example, dipping the umbilicus in a 7% iodine tincture at birth showed no effect in reducing the incidence of umbilical infections [58], suggesting that it may not be effective. Risk factors for umbilical infection are debated in the literature but include bedding type [59] and FTPI [8,60]. Additionally, Van Camp et al. [58] identified an association between umbilical infections and timing of colostrum feeding, with calves that had delayed colostrum feeding having higher odds of infection. Effective colostrum management, critical to preventing preweaning morbidity and mortality, will be discussed in detail in the next section.

## 6. Colostrum Management

Calves are born agammaglobulinemic [61] and depend on TPI from their dam via colostrum to establish early immunity [54]. The five CCPs of colostrum management, quantification, quickness, quantity, quality, and cleanliness, are crucial to ensure successful transfer of passive immunity.

### 6.1. Quantification

Quantification refers to quantifying the total absorbed colostral IgGs. This is the initial step in assessing colostrum management, offering valuable insight into whether the current practices are sufficient or require improvement. Assessing the quantification of passive immunity can be achieved by directly measuring IgG with radial immunodiffusion, or through indirect measures, such as using a Brix or total protein refractometer. Using a refractometer to evaluate STP is a rapid, inexpensive, and convenient method for estimating serum IgG levels [62]. While STP can be measured from 24 h to 9 days after colostrum feeding, there is reduced agreement with serum IgG beyond 72 h [63], and thus capturing this information earlier can help improve the accuracy of TPI classification. Another important consideration is that the agreement between STP and serum IgG is poorer for calves fed colostrum replacer powder than those fed maternal colostrum [64].

**Figure 1:**
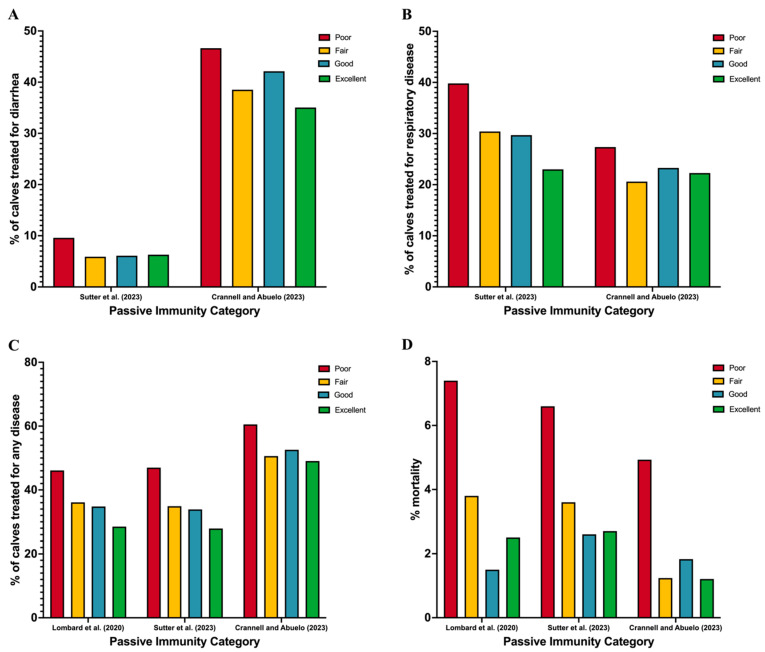
Proportion of calves with (**A**) diarrhea, (**B**) respiratory disease, (**C**) any preweaning disease, and (**D**) mortality dependent on the category of transfer of passive immunity (poor to excellent) using data from Lombard et al. [67], Sutter et al. [68], and Crannell and Abuelo [69].

Traditionally, FTPI was based on 10 g/L IgG [65], which corresponded to the dichotomous cut-points of 5.2 g/dL or 5.5 g/dL, depending on the desired sensitivity and specificity [66]. However, Lombard et al. [67] created a consensus document suggesting that the routine monitoring of serum total proteins and serum IgG should instead classify calves into the categories of poor, fair, good, and excellent, with proposed targets for the proportion of calves in each category (Table 2), rather than using a dichotomous cut-point. Compared with calves in the excellent category, those in the poor category have an increased risk for NCD, BRD, and mortality (Figure 1). Additionally, Lopez et al. [64] determined the STP cut-off for FTPI when whole bovine dried colostrum replacer was fed at 4.9 g/dL; however, these results should not be extrapolated to other replacer products due to differences in manufacturing and nutritional compositions. By assisting producers in optimizing the remaining four CCPs of colostrum management, the proportion of calves in the excellent category should be increased.

### 6.2. Quickness

Quickness refers to both the amount of time elapsed between calving and colostrum collection, as well as the time between birth and colostrum administration. Fischer et al. [70] reported that delaying colostrum feeding by 6 and 12 h after birth decreased the maximum apparent efficiency of IgG absorption, leading to decreased TPI compared with calves fed colostrum immediately after birth. Additionally, the IgG content is highest in colostrum collected soon after calving and declines by 3.7% with each passing hour post-partum, although the exact mechanism for its decline has yet to be elucidated [71]. Therefore, it is important both to rapidly harvest colostrum and quickly feed colostrum to maximize TPI.

### 6.3. Quantity

General recommendations are to feed calves 300 g of IgG within 2 h of birth [8,70], which is the quantity of IgG required to achieve excellent TPI, as defined by Lombard et al. [67]. The first colostrum feeding should be 8.5% to 10% of bodyweight [72], and feeding <2.5 L should be discouraged, as it is associated with increased odds for FTPI [73]. Beyond the first colostrum feeding, multiple feedings of colostrum have been reported to increase serum IgG concentrations [74] and feeding >5.9 L in the first 24 h of life is associated with higher STP [75]. Further, calves that receive two feedings of colostrum have been associated with lower odds of FTPI, greater preweaning ADG, and a lower probability of NCD and BRD [76]. Multiple feedings are also beneficial if calves are fed a lower mass of IgG at the first feeding, as adding in two additional feedings at 6 and 12 h can result in similar TPI as that of calves fed higher IgG doses at the first feeding [77].

### 6.4. Quality

While colostrum contains a multitude of bioactives and immune cells important to the health and development of calves [78], assessing the quality of colostrum traditionally refers to the IgG content. The industry standard cut-point for good-quality colostrum is 50 g/L IgG [8]. However, it is important to note that this cut-off point is based on calves achieving serum IgG levels ≥ 10 g/L [79]. With revised recommendations to maximize the proportion of calves with serum IgG ≥ 25 g/L [67], this cut point is likely too low. Specifically, for every 10 g/L increase in colostral IgG, there is an associated 1.1 g/L increase in serum IgG [80], which highlights the importance of feeding high-quality colostrum. The quality can be evaluated on-farm using a digital or optical Brix refractometer [81], where the level of IgG is highly correlated with Brix % and 22% Brix indicates 50 g/L of IgG [81]. To ensure accurate Brix measurements, the refractometer should be frequently calibrated using distilled water to obtain a reading of 0% in the case of an optical refractometer or by pressing the zero button on digital refractometers with distilled water in the well. Quality can also be assessed using colostrometers, which also has good agreement with colostral IgG concentrations, where a cut point of 80 g/L is indicative of 50 g/L of IgG [82]. It is important to note that colostrometers require colostrum be at 22 °C to obtain an accurate measurement [82].

When faced with low colostral Brix %, prepartum factors should be investigated. Specifically, cows fed controlled energy diets to meet energy requirements have higher colostral IgG concentrations than cows overfed energy during the dry period [83]. Further, prepartum diets with neutral detergent fiber ≤ 39.0% DM and low to moderate DCAD (≥−15.9 mEq/100 g) are associated with increased colostral IgG [84]. Lastly, a lower starch prepartum diet (≤18.5%) was also associated with increased colostral IgG in primiparous animals [84]. Nonetheless, low Brix % colostrum can be enriched using colostrum replacer powder to improve its quality. Specifically, adding approximately 145 g of colostrum replacer per liter of colostrum directly to the low-quality colostrum (30 g/L IgG, 15.8% Brix) was reported to improve it to good-quality colostrum (60 g/L IgG, 26.4% Brix), and resulted in similar levels of TPI compared to calves fed maternal colostrum containing 60 g/L IgG [85]. However, it is important to note that there was no effect on serum IgG concentrations when enriching beyond 60 g/L IgG [85].

### 6.5. Cleanliness

A higher total bacteria count in colostrum is associated with reduced STP and plasma IgG, as well as decreased efficiency of IgG absorption [86]. Thus, colostrum should be fed or stored in a fridge or freezer immediately, as storing colostrum at room temperature promotes bacterial proliferation, which can lower serum IgG in calves compared to those fed refrigerated colostrum [87]. Cummins et al. [87] speculated that bacteria may bind to the IgG, altering its structure and rendering it unable to bind to sites at the intestinal wall, thus reducing TPI. Refrigerated colostrum should be used within 2 days [88] unless preservatives, such as potassium sorbate, are added. A final solution of 0.5% (*w*/*v*) potassium sorbate can extend the refrigeration time to 6 days, which can be achieved by adding 10 mL of 50% potassium sorbate per liter of colostrum [88]. If colostrum is stored in the freezer, it is essential to thaw it at temperatures < 60 °C to minimize the denaturation of IgG [89]. Storing frozen colostrum in bags increases the surface area, allowing for faster thawing compared to storing in bottles. Additionally, sous vide devices may be used to thaw colostrum, as it maintains and circulates the water bath at a consistent temperature.

Automated milking systems have different CCPs where colostrum contamination may occur, and becoming familiar with these areas can be achieved through discussion with brand representatives. General areas to consider include hoses and nozzles associated with colostrum collection, as well as the collection buckets. Once these CCPs are identified, assessing the hygiene of these areas can reduce the risk of contamination. Cleanliness can be assessed at various points throughout the colostrum harvest and feeding processes by collecting a series of colostrum samples for total aerobic and coliform bacteria counts to determine the point of contamination. The recommended maximum bacterial thresholds for fresh colostrum are 100,000 cfu/mL total bacteria count and 10,000 cfu/mL coliform [79]; however, it is important to note that these thresholds are based on expert opinion.

### 6.6. Feeding Method

While not one of the traditional five CCPs of colostrum management, the feeding method should be considered. Feeding colostrum via a nipple bottle or an esophageal tube does not affect the absorption of IgG [90]. However, allowing calves to suckle from the dam should be avoided, as it is associated with reduced TPI compared to hand-fed calves due to inadequate colostrum intake [91].

## 7. Preweaning Nutrition

Nutrition influences the immune system and health of calves. When investigating calf health issues, it is essential to assess whether transition milk is fed, the volume and type of milk fed (e.g., bulk tank milk, waste milk, milk replacer (MR), acidified milk, or pasteurized milk), the amount and type of starter offered, the availability and quality of water, and hygiene practices during feeding.

### 7.1. Transition Milk

Transition milk refers to milkings 2 to 6 after calving [92] and may be a tool to help reduce the incidence of NCD. Feeding transition milk for the first 3 days of life improves gastrointestinal mass and villus development [93], as well as IgG persistency [74], all of which may improve nutrient absorption and intestinal health. Feeding transition milk has been associated with fewer antimicrobial treatments in calves [94] and improved preweaning average daily gain (ADG) [95]. Further, feeding transition milk has positive effects on calf health and growth if fed for a duration of three days [95] up to three weeks [96]. If the collection of transition milk is not feasible, colostrum replacer powder can be used to mimic transition milk using a 50–50 blend of milk and colostrum replacer powder [97]. Additionally, colostrum replacer powder can be used longer-term to reduce morbidity. Specifically, supplementation of MR with 150 g of colostrum replacer powder twice daily for 14 days is associated with reduced NCD, BRD, and antimicrobial usage [98]. Similar results have also been reported when using 70 g of colostrum replacer twice daily for 14 days [99].

### 7.2. Plane of Nutrition

Calves fed lower planes of nutrition have less effective immune systems than those fed higher planes. A low plane of nutrition refers to feeding rates of 300 to 450 g/d of milk or MR solids for Jerseys and 400 to 600 g/d for Holsteins, while high rates of feeding are defined as >700 g/d and 900 g/d solids for Jerseys and Holsteins, respectively [42]. Calves fed low planes of nutrition have impaired neutrophil oxidative burst [100] and experience more severe pathophysiological responses to disease, resulting in increased inflammation and mortality [101]. Low planes of nutrition may also impair or delay the development of the adaptive immune response in calves [101]. This may explain why feeding higher planes of nutrition is associated with a lower prevalence of BRD [102] and faster recovery after *Cryptosporidium parvum* challenge [103].

A common rationale for feeding restricted amounts of milk is to stimulate early intake of starter; however, the efficiency of ME use is lower for calf starter than that of milk components [42]. This highlights the importance of milk or MR delivering the majority of nutrients to preweaned calves. Despite NASEM [42] defining high planes of nutrition for Holstein calves as >900 g/d of milk or MR solids (corresponding to 6 L/d when feeding at 13% total solids), calves offered <8 L/d display signs of hunger [104]. When calves are offered higher volumes of milk (10 and 12 L/d), they gain more during the preweaning phase and maintain the weight advantage during and after weaning, with no difference in postweaning starter intake [104]. By knowing the ME provided per DM kilogram of milk or MR, as well as the energy requirements of the calf, you can determine if the plane of nutrition is appropriate. This can be assessed by using the following calculations [42]:

Whole milk ME:

Mcal/kg = [(0.057 × CP % + 0.094 × 0.945 crude fat % + 0.0395 × lactose %) × 0.93]/(1 − moisture)

Milk replacer ME:

Mcal/kg = [(0.057 × CP % + 0.094 × 0.945 crude fat % + 0.0395 × lactose %) × 0.91]/(1 − moisture)

Table 3 illustrates the daily energy and protein requirements of Holstein calves, determined by their body weight and target gain, considering only milk or MR intake. Given that calves will also be consuming starter in varying amounts based on age and milk allowance, part of the calf’s ME requirements will be met by starter intake, which is not considered in Table 3. It is important to note that extra energy is required when temperatures are above or below the thermoneutral zone of calves (Table 4), which is 15–25 °C for calves younger than 3 weeks and 5–25 °C for calves older than 3 weeks [42]. Increasing caloric intake can be achieved through increased volume [105], additional feedings [106], or increased total solids of milk [107].

Regular assessment of total solids of milk replacer and whole milk, as well as calibration of automated milk feeders (AMFs) are important CCPs, as feeding milk with <10% total solids has been associated with an increased prevalence of BRD [108]. When water is provided ad libitum, total solids can be safely increased to 20% without negative effects [109]. When assessing the total solids in MR using a Brix refractometer, an adjustment factor must be applied. For optical refractometers, a value of 1.08 must be added to the Brix reading and 1.47 added for digital refractometers [110].

### 7.3. Milk Sources

Whole milk is the predominant liquid diet type fed to calves in the United States and Ontario, Canada, followed by MR [111,112]. Compared to whole milk from Holsteins, MR often contains more lactose (40–50% vs. ~35% DM basis; [113]), less fat (16–20% vs. ~30%), and equivalent protein levels (20–24% vs. ~24%; [114]). Depending on the MR, whole milk may contain 10 to 20% more energy at comparable volumes due to its higher fat content [114] and greater digestibility [42,115,116]. However, in most cases, whole milk does not provide sufficient selenium and needs to be supplemented [42,117].

Both animal- and plant-derived ingredients have been used to formulate MR. Research regarding the health implications of different MR fat sources is required; however, in calves fed ad libitum, MR with only animal-derived fat sources increased MR intake and improved preweaning gain compared to mixed or plant-derived sources [118]. Regarding protein sources in MR, non-milk proteins may have incomplete amino acid profiles and lower digestibility than milk protein sources [119,120]. As protein digestion of calves in the first 2–3 weeks of life is immature and poor, even with high-quality protein sources [121], opting for MR with milk proteins may help increase protein digestibility and nutrient availability in young calves [119,122].

The ideal macronutrient profile of the MR is dependent on the total ration (i.e., the starter and volume of milk fed), as well as the target weight gain. However, feeding high-fat MR (≥23%) is associated with decreased mortality [6], better fecal consistency [113], and fewer disease events requiring therapeutic interventions [123]. Thus, feeding high-fat MR could be prioritized, especially when calves are feed-restricted or at increased risk of disease and environmental stressors (e.g., cold stress).

While feeding waste milk is a common practice in the United States [111], it has been associated with higher levels of NCD and altered fecal microbiomes [124], as well as a higher proportion of resistant fecal *E. coli* isolates [125] than calves fed bulk tank milk. Therefore, feeding waste milk should be discouraged.

### 7.4. Calf Starter

Calf starter should be provided in the first week of life, as it is important for the development of the rumen [126]. When evaluating the macronutrient profile of a calf starter, it needs to be considered as part of the total diet of the calf and not as a stand-alone feedstuff. Thus, the plane of milk feeding needs to be considered. Calves fed lower quantities of milk will consume more starter than calves fed high quantities of milk [127] and may benefit from low-starch pelleted starters (≤25%) compared with high-starch texturized starters (≥40%) [128]. Additionally, fine particles should be avoided, especially when forages are not provided, to prevent bloat [42]. At least 80% of the starter should have a particle size of >1190 μm, regardless of the physical presentation of the starter, which can be examined by wet-sieving techniques [42]. Regarding forage provisions, forage may improve feed intake and growth in addition to stimulating rumen development and reducing nonnutritive oral behaviors [129]. Further, forages support rumen pH and reduce the risk of acidosis [129]. Ideally, calves should be offered a mixed ration of forages and starter, as feeding forages separately results in variability in forage intake [130]. There is no consensus regarding how much forage should be provided, as optimal amounts depend on the particle size and fermentability of the ration; however, most literature supports no more than 15% of total DMI coming from forages [131]. Additionally, calves offered forages in the form of grass hay have greater total DMI, starter intake, and ADG during weaning compared to calves offered a silage-based total mixed ration [132]. As calves have smaller ruminal capacity, the higher moisture content of silages may increase gut fill to subsequently reduce intakes before and during weaning [132] and thus should be avoided.

### 7.5. Water

The availability of water is important to encourage starter intake [133]. Specifically, compared to calves that received water at day 28, calves offered water from birth had a 30% increase in starter intake and gain [133]. Further, Wickramasinghe et al. [134] reported that providing free-choice water from the first day of life increased milk intake by 300 g/d and improved growth compared to calves offered water at 2 weeks of age [134]. In the first week of life, calves can consume as much as 1.5 L/d [135] and providing warm (16 °C to 18 °C) compared to cold (6 °C to 8 °C) water results in greater water intake [136]. Calves will exponentially increase their water intake with increasing ambient temperature, with consumption predicted to exceed 3 L when temperatures reach 30 °C [137], highlighting the need for increased water provision in heat-stressed calves [42].

Submitting water samples for mineral and bacteriological analysis may aid in morbidity and mortality investigations. Excess iron can allow for bacterial proliferation [138] and may induce or exacerbate diarrhea. Additionally, caution should be taken when offering fluids with osmolarities > 600 mOsm/kg, as it may lead to reduced calf health [139] and digestive upset [110]. Feeding water with high total dissolved solids may exacerbate digestive upsets, especially as softened water often contains elevated sodium levels, which can contribute to hyperosmolarity. This may be of particular concern for calves fed milk with a high percentage of total solids.

### 7.6. Hygiene Practices

Hygiene can influence calf health, as feeding high-bacterial-count milk is associated with increased prevalence of NCD [108] and worsened health scores [140]. Feeding equipment hygiene can be assessed using ATP-luminometers, as they have a strong positive correlation with total bacteria count [141]. Important CCPs to sample include colostrum harvest and delivery equipment, bottles/buckets, nipples, esophageal feeders, milk taxis, and hoses of AMFs. If poor hygiene is identified, it is important to investigate the farm’s cleaning SOPs and equipment age. While literature to support cleaning methods for calf feeding equipment is scarce, we recommend cleaning feeding equipment similarly to how bulk tanks are cleaned. Specifically, soaking and scrubbing equipment using a chlorinated alkalinizing detergent (at concentrations and temperatures recommended by the manufacturer) to break down milk fats and biofilms, followed by an acid (i.e., phosphoric acid), should be conducted for sanitation and removal of water and milk salts.

## 8. Housing and Environment

### 8.1. Group Size and Stocking Density

Industry recommendations are to provide calves with a minimum of 3.3 m^2^ (35 ft^2^) per calf [142]; however, in group housing systems, providing calves with more space may further improve health independent of group size [140]. Specifically, Jorgensen et al. [140] reported that for every additional square meter of space, there were decreased odds of clinical signs of respiratory disease. Regardless of space per calf, increased group size is associated with a higher prevalence of BRD [143], with improved health outcomes associated with group sizes of less than seven or eight calves [143,144]. In pair-housed calves, no differences in morbidity have been detected compared with individually housed calves [145,146]. Additionally, calves paired early (within a week of birth) have higher starter intake and ADG than late-paired (6 weeks) or individually housed calves [147].

An additional risk factor to consider is that housing young calves with older cattle is associated with increased mortality [148], and higher levels of NCD and BRD [108]. Thus, keeping the age range of cattle housed in shared air as close as possible may help reduce morbidity and mortality.

### 8.2. Bedding

Providing clean, dry bedding is important to calf health. Medrano-Galarza et al. [108] reported a lower prevalence of NCD in pens where fresh bedding was added more frequently (every 2 to 3 days) compared to those where bedding was replaced less often (every 7 to >10 days). Further, a deeper wet bedding pack was associated with a higher prevalence of BRD [108], and recommendations are to provide at least 7.6 cm of dry bedding [149]. Bedding type is also associated with morbidity. Specifically, calves bedded on long straw have lower mortality [150] as well as the fewest days of diarrhea and lower bedding coliform counts [151]. The dustiness of the bedding is also another important consideration [102]. Dustier bedding substrates have been associated with an increased risk of BRD by exposing calves to fine particulate matter, which will be discussed further in the next section.

As temperatures drop below the critical threshold, calves will use metabolic energy to keep warm [42]. Bedding helps calves retain body heat and can be evaluated through a nesting score, which measures how well a calf can settle into the bedding when lying down. Nesting scores are based on leg visibility and scored as 1 (legs fully exposed), 2 (legs partially visible), and 3 (legs not visible) [152]. The prevalence of respiratory disease is lower as nesting score increases [152] and bedding with long straw can assist in achieving high bedding scores.

### 8.3. Air Quality and Ventilation

Poor air quality is associated with the development of BRD and should be assessed in indoor-housed calves, especially when BRD prevalence is high. Fine particulate matter (≤1 μg/m^3^), measurable using air quality sensors, is associated with increased odds of lung consolidation and the presence of respiratory pathogens [153]. Dust control strategies, such as using low-dust beddings and water trucks to spray surrounding roads, can improve respiratory health [102]. Prolonged exposure to ammonia levels > 4 mg/L also increases the odds of lung consolidation [154]. While there are no recommendations regarding optimal humidity for naturally ventilated barns, increased relative humidity at the pen level is associated with poorer calf health scores [154]. If pen humidity is higher than ambient humidity, improvements should be made through better ventilation and drainage.

While a complete overview of ventilation assessments is beyond the scope of this article, general ventilation rate recommendations are to have a minimum of four air exchanges per hour year-round [155], whereas in the summer, increasing ventilation rates can aid in heat abatement, and recommendations are to have at least forty air exchanges per hour [155]. While providing fresh air is important for calf health, draughts should be avoided, as air velocities > 0.8 m/s increase the risk of lung consolidation [154]. Draughts are generally considered present at > 0.3 m/s [156] and air velocity can be assessed by using an anemometer at calf level.

Heat stress can be determined by examining respiration rates, and >40 breaths per minute are an indication of heat stress in preweaned calves [157]. Additional heat abatement strategies include the installation of an 80% shade cloth over outdoor hutches and fans in indoor-housed calves. Data regarding the effects of heat abatement on growth and feed efficiency are mixed [158,159]. However, tendencies for reduced treatment events have been reported in cooled calves compared to heat-stressed calves [158], but further research is required to assess the effects of heat abatement strategies on morbidity.

### 8.4. Drainage

Calves produce a large amount of liquid waste due to their predominantly liquid diets, and drainage is important for keeping bedding dry and reducing humidity and ammonia. Adequate drainage can be accomplished using tiled gravel beds 0.5 m deep below the bedded area, or by grading solid flooring to have a 2% slope toward a gutter [142].

## 9. Vaccination

Vaccination of the preweaned calf can pose a challenge due to high levels of maternal antibodies resulting in delayed antibody production by the neonatal calf [160]. However, intranasal vaccines administered to preweaned calves in a field trial reduced the odds of lung consolidation [161]. Further, in high-risk veal calves, the use of parenteral and intranasal vaccines upon arrival has been reported to reduce the odds of developing chronic BRD, improve long-term cure of BRD after treatment, and reduce the incidence of new pneumonia cases [162]. Thus, implementing vaccine strategies can help to reduce morbidity in preweaned dairy calves and should be considered, especially in high-risk scenarios.

## 10. Calf Health Records Analysis and Feedback

Complete and accurate calf health records are crucial for effective health investigations and enable the evaluation of management practices and protocol changes. Calf health record accuracy may be improved by using standardized health scoring systems for objective disease diagnosis. Specifically, the Wisconsin Calf Health Scoring Chart [163] and the UC Davis Bovine Respiratory Disease Scoring System [164] can help to positively identify calves with BRD, and using fecal consistency scoring [165] can help to objectively identify calves with NCD. Unfortunately, challenges exist in terms of data capture and analysis. Atkinson [166] reported that only 39% of Welsh dairy producers recorded any calf illnesses and Uyama et al. [94] found that only half of Canadian dairy farms had complete treatment calf health records in a format that could be analyzed. Additionally, almost half of producers reported that their veterinarians did not provide recommendations based on their calf health records [167]. As producers seek practical, customized recommendations based on their calf health data [168], veterinarians who offer analysis and feedback on these records will have producers that are more likely to consistently record the primary data [167]. Motivating on-farm change can be a challenge, but effective methods to motivate improvements in calf care include SOPs, assessing calves, and goal setting [169]. Further, producers report improved SOP utilization and compliance when veterinarians are involved in their development [50].

Calf health record completeness may be improved by facilitating electronic recording of data, keeping records in close proximity to calves, and providing analysis and feedback [167]. There are many herd management software packages available that can facilitate data capture, but an alternative is creating an online spreadsheet that can be accessed by all calf workers from their mobile devices. This allows employees to record data calf-side and provides an efficient format for analysis.

When configuring these spreadsheets for data capture, KPIs to monitor include TPI, incidences and treatments (product, dose, duration, and route) of NCD and BRD, mortality rate, prevalence of lung consolidation (determined by thoracic ultrasound), and ADG. Although this review has primarily focused on disease management, ADG is an important metric to monitor in preweaned calves, as it is positively associated with future milk production [4] and negatively impacted by morbidity [5]. A check list of KPIs can be found in Table 5.

## 11. Conclusions

Calf health investigations are complex due to the multifactorial nature of disease development. However, by thoroughly examining the various aspects of calf care and analyzing calf health records, veterinarians can more effectively identify issues at CCPs and offer practical, customized solutions for their dairy clients.

## Figures and Tables

**Table 2:** Serum IgG concentrations and corresponding serum total protein (STP) and Brix measurements for each transfer of passive immunity (TPI) category and suggested target proportion of calves ^a^.

TPI Category	Serum IgG (g/L)	Equivalent STP (g/dL)	Equivalent Brix %	Target % of Calves
Excellent	≥25.0	≥6.2	≥9.4	>40
Good	18.0–24.9	5.8–6.1	8.9–9.3	~30
Fair	10.0–17.9	5.1–5.7	8.1–8.8	~20
Poor	<10.0	<5.1	<8.1	<10

^a^ Adapted from Lombard et al. [67].

**Table 3:** Daily energy and protein requirements ^a^ of Holstein calves fed only milk or milk replacer.

Bodyweight (kg)	Target ADG ^b^ (g/d)	ME ^c^ (Mcal/d)	MP ^d^ (g/d)	CP ^e^ (% of DMI)
50	600	3.98	193	23.6
	900	5.13	267	25.2
75	900	6.10	284	22.6
	1200	7.40	360	23.6
100	900	6.93	300	21.0
	1200	8.31	379	22.0
125	900	7.70	316	19.9
	1200	9.14	397	21.0

^a^ Adapted from NASEM, 2021 [42]. ^b^ Average daily gain. ^c^ Metabolizable energy. ^d^ Metabolizable protein. ^e^ Crude protein.

**Table 4:** Percentage increase in metabolizable energy ^a^ required for maintenance in young calves (<3 weeks of age) ^b^ based on ambient temperature.

Ambient Temperature (°C)	Ambient Temperature (°F)	Percentage Increase in Metabolizable Energy
30	86	+9%
20	68	0%
10	50	+19%
0	32	+38%
−10	14	+56%
−20	−4	+75%
−30	−22	+94%

^a^ Adapted from NASEM, 2021 [42]. ^b^ Based on a calf with empty body weight of 45 kg.

**Table 5:** Key performance indicators (KPIs) and suggested targets for monitoring preweaning dairy calf health management.

Key Performance Indicator	Target
Transfer of passive immunity ^a^	≥40% excellent ~30% good ~20% fair <10% poor
Neonatal calf diarrhea morbidity rate ^b^	<15%
BRD morbidity rate ^b^	<10%
Mortality rate ^b^	<3%
Lung consolidation ^c^ (≥3 cm^2^) at weaning	<15%
Average daily gain ^b^	≥800 g/d

^a^ Derived from Lombard et al. [67]. ^b^ Derived from Dairy Calf and Heifer Association 2023 Gold Standards [170]. ^c^ Derived from WeanClean [171].

## Data Availability

Data are contained within the article.
